# Coexistence of Relapsing Polychondritis and Crohn’s Disease: Clinical Insights from a Rare Case

**DOI:** 10.3390/jcm15041609

**Published:** 2026-02-19

**Authors:** Sang Wan Chung

**Affiliations:** Division of Rheumatology, Department of Internal Medicine, School of Medicine, Kyung Hee University, Seoul 02453, Republic of Korea; wanyworld83@gmail.com; Tel.: +82-2-958-8200

**Keywords:** relapsing polychondritis, Crohn’s disease, inflammatory bowel diseases

## Abstract

Relapsing polychondritis (RP) is a rare immune-mediated inflammatory disorder characterized by recurrent inflammation of cartilaginous structures. Although RP frequently coexists with other autoimmune disorders, its association with inflammatory bowel disease (IBD), particularly Crohn’s disease, has been rarely described. We report the case of a 53-year-old man who presented with bilateral auricular inflammation sparing the earlobes and was diagnosed with RP based on clinical and histopathological findings. During treatment with systemic corticosteroids and methotrexate, he developed severe abdominal pain accompanied by inflammatory arthritis. These symptoms were initially considered related to treatment; however, subsequent endoscopic and histologic evaluation revealed Crohn’s disease involving the terminal ileum. Therapeutic adjustment, including discontinuation of nonsteroidal anti-inflammatory drugs and optimization of immunosuppressive therapy, resulted in resolution of both gastrointestinal and musculoskeletal symptoms. This case emphasizes the importance of considering concomitant IBD in patients with RP who develop unexplained gastrointestinal manifestations. Recognizing this rare coexistence may facilitate earlier diagnosis and more appropriate therapeutic decision-making in patients with multisystem inflammatory disease.

## 1. Introduction

Relapsing polychondritis (RP) is a rare immune-mediated systemic inflammatory disorder characterized by recurrent inflammation and progressive destruction of cartilaginous structures, most commonly affecting the auricles, nasal cartilage, and respiratory tract [1]. Auricular chondritis with sparing of the earlobes represents the most characteristic clinical manifestation and frequently serves as a key diagnostic clue [1,2]. Because RP lacks disease-specific laboratory markers and presents with heterogeneous clinical features, diagnosis is often delayed or overlooked, particularly in the early stages of the disease [2].

Approximately 30% of patients with RP have concomitant autoimmune or inflammatory disorders [2]. However, coexistence with inflammatory bowel disease (IBD), particularly Crohn’s disease (CD), is exceedingly rare and has been documented in only a limited number of case reports [3,4,5]. Although extraintestinal manifestations are common in IBD, cartilaginous inflammation consistent with RP represents one of the rarest associations [6].

The clinical overlap between RP and CD poses diagnostic challenges, as both conditions are characterized by episodic systemic inflammation and may respond to immunosuppressive or biologic therapies [3,5]. Moreover, gastrointestinal symptoms that develop during treatment for RP may be misattributed to medication-related adverse effects, potentially delaying the recognition of underlying IBD [5]. Given the rarity of this coexistence and the lack of standardized management strategies, additional case reports are needed to enhance clinical awareness and to improve diagnostic and therapeutic decision-making.

Herein, we report a rare case of RP coexisting with CD and discuss its clinical implications.

## 2. Case Report

Case presentation: A 53-year-old man with a 6-month history of bilateral auricular redness and tenderness, sparing the earlobes, was referred to our rheumatology clinic. The patient reported no peripheral joint symptoms except for neck and anterior chest wall pain. He had no significant past medical history and was not taking any medications.

**Figure 1:**
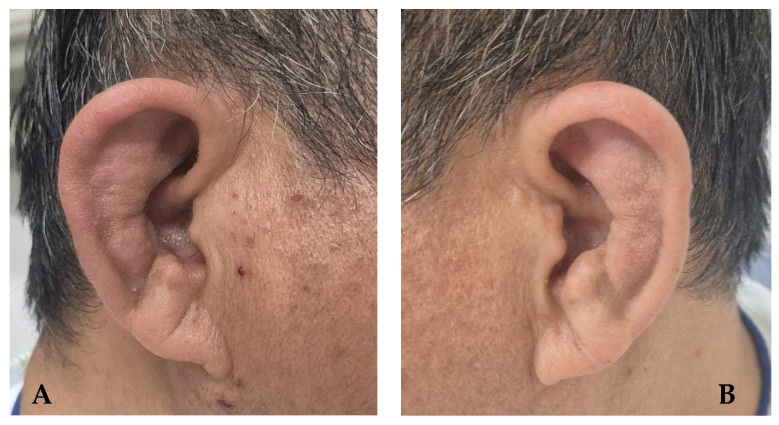
Photographs of both ears demonstrating bilateral auricular erythema with sparing of the ear lobes ((**A**), right; (**B**), left), consistent with bilateral auricular chondritis.

Initial diagnostic evaluation: Laboratory testing revealed elevated inflammatory markers, with a high-sensitivity C-reactive protein level of 0.39 mg/dL (reference range, <0.10 mg/dL), and an erythrocyte sedimentation rate of 74 mm/h (reference range, <20 mm/h). Antinuclear antibody was positive at a low titer (1:40, speckled pattern), whereas anti-cyclic citrullinated peptide antibody and rheumatoid factor were negative. Human leukocyte antigen B27 was positive. Complete blood count, serum electrolytes, and other biochemical parameters were within normal limits. Radiographs of the sacroiliac joint were unremarkable. On physical examination, bilateral auricular swelling and tenderness with sparing of the earlobes were observed (Figure 1), along with tenderness over several sternocostal joints. An auricular biopsy demonstrated mixed inflammatory cell infiltrate with blurring of the interface between the perichondrium and the cartilage and extension throughout the cartilage. Based on chronic auricular chondritis, sternal chondritis, and supportive histopathological findings, the patient was diagnosed with RP according to the diagnostic criteria proposed by Damiani and Levine [7].

Initial treatment and clinical course: Initial treatment consisted of low-dose oral prednisolone (20 mg/day), methotrexate (10 mg/week), and nonsteroidal anti-inflammatory drugs (NSAIDs) at the outpatient clinic. One month later, the patient presented to the emergency department with a 1-week history of severe periumbilical abdominal pain and bilateral knee pain with swelling. He denied diarrhea or hematochezia. Believing the symptoms were medication-related, he had discontinued his prescribed corticosteroids for 1 week prior to presentation to the emergency department.

**Figure 2:**
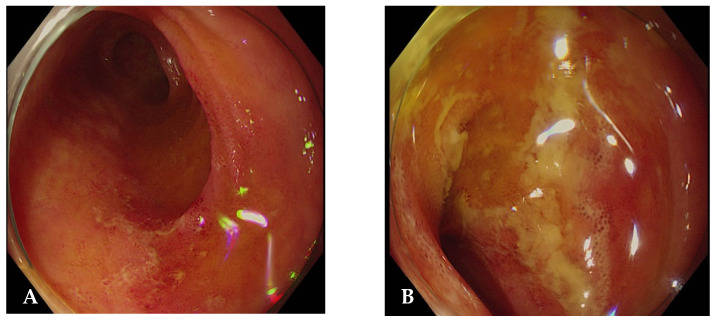
Colonoscopic findings showing multiple inflammatory erosions in the terminal ileum (**A**,**B**).

Reevaluation and gastrointestinal diagnosis: At presentation, infectious etiologies were considered; however, the patient had no fever, diarrhea, hematochezia, leukocytosis, or recent antibiotic exposure. Given the low clinical suspicion for infectious colitis, including Clostridioides difficile infection, further evaluation focused on inflammatory causes, and colonoscopy was performed. Colonoscopy revealed multiple inflammatory changes with skip lesions in the terminal ileum, consistent with CD (Figure 2). Colonoscopy demonstrated inflammatory lesions confined to the terminal ileum without colonic involvement, consistent with Montreal classification L1 disease. Histopathological examination of the ileal biopsy demonstrated marked inflammatory cell infiltration without granuloma formation, which was comparable to the findings in CD. The fecal calprotectin level was markedly elevated at 640 μg/g (reference range, <50 μg/g). The patient was therefore diagnosed with coexisting RP and CD.

**Figure 3:**
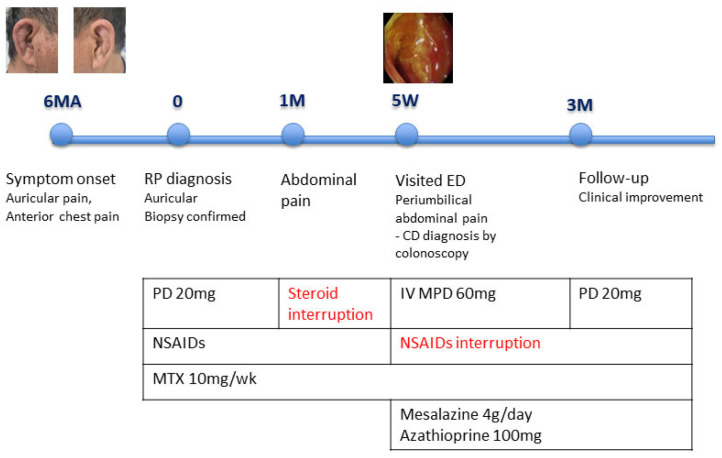
Timeline chart illustrating the patient’s medication history and major clinical events; MA: months ago; M: months; W: weeks; ED: Emergency Department; PD: prednisolone; IV: intravenous; MPD: methylprednisolone; NSAIDs: Non-steroidal anti-inflammatory drugs; MTX: methotexate.

Therapeutic adjustment and follow-up: NSAID therapy was discontinued, and treatment was initiated with mesalazine (4 g/day) and intravenous methylprednisolone (60 mg/day). High-dose corticosteroid therapy resulted in marked improvement of both abdominal pain and arthralgia. Mesalazine was selected as initial therapy given the localized ileal involvement, absence of stricturing or penetrating complications, and the need for cautious treatment escalation in the setting of concomitant immunosuppressive therapy for relapsing polychondritis. At 3 months of follow-up, the patient remained symptom-free while receiving a tapered dose of prednisolone (20 mg/day) and azathioprine (Figure 3).

## 3. Discussion

RP is a rare immune-mediated systemic inflammatory disease characterized by recurrent inflammation and progressive destruction of cartilaginous tissues, with a highly variable clinical course and unpredictable organ involvement [1,2]. Auricular chondritis with sparing of the earlobes remains the most distinctive and frequently reported manifestation and often provides the earliest diagnostic clue [8]. Nevertheless, the absence of disease-specific serologic or histopathological markers makes RP a diagnostic challenge, particularly in patients presenting with multisystem involvement or overlapping inflammatory conditions [2]. Recent reviews emphasize the expanding clinical spectrum of relapsing polychondritis, which involves complex immune mechanisms and diverse phenotypes that may overlap with other systemic inflammatory disorders. This improved understanding may assist clinicians in recognizing atypical presentations and associated comorbidities [9]. 

The coexistence of RP and CD is exceptionally rare. Although extraintestinal manifestations occur in up to 40% of patients with IBD, cartilaginous involvement consistent with RP has been reported only sporadically [6,10]. Most reported cases describe isolated auricular or nasal chondritis, often leading to diagnostic uncertainty regarding whether RP represents a distinct comorbid autoimmune disorder or an atypical manifestation within the spectrum of IBD-related systemic inflammation [3]. Current evidence supports RP as a separate disease entity, as its target tissues, clinical behavior, and diagnostic criteria differ substantially from conventional extraintestinal manifestations of IBD [3,8].

From an immunopathogenic perspective, both RP and CD are immune-mediated disorders characterized by dysregulated innate and adaptive immune responses. In RP, immune reactivity against cartilage matrix components, including type II collagen and proteoglycan-rich structures, has been implicated in disease pathogenesis [11]. In contrast, CD is driven by chronic intestinal inflammation involving aberrant T-cell activation, intestinal barrier dysfunction, and dysregulated cytokine signaling [10,12]. Despite these mechanistic differences, tumor necrosis factor-α (TNF-α) plays a central role in both diseases, promoting systemic inflammation and tissue damage [13]. This shared cytokine pathway provides a biologically plausible rationale for the observed therapeutic efficacy of TNF-α inhibitors in patients with coexisting RP and CD.

The temporal relationship between RP and CD varies widely across reported cases. RP may precede, coincide with, or follow the diagnosis of CD, indicating the absence of a consistent sequence [4,8]. In the present case, gastrointestinal symptoms emerged during glucocorticoid tapering for RP, initially raising concern for treatment-related adverse effects. However, persistent abdominal symptoms and confirmatory endoscopic and histopathological findings ultimately established the diagnosis of CD. This clinical scenario highlights an important diagnostic pitfall: gastrointestinal manifestations arising during RP treatment should not be automatically attributed to medication effects, particularly when symptoms persist or worsen despite dose adjustment [4,14].

The differential diagnosis in such cases is broad and includes medication-induced enteropathy, nonspecific inflammatory changes, and classical extraintestinal manifestations of IBD. Awareness of the rare coexistence of RP and CD is therefore essential to prevent diagnostic delay. Early recognition is particularly important given that untreated CD may progress to irreversible bowel damage and systemic complications [10,12].

Therapeutic strategies for patients with coexisting RP and CD remain largely empirical, as randomized controlled trials are lacking. Systemic corticosteroids are often effective for acute disease control in RP but are associated with frequent relapse during tapering and significant long-term adverse effects [2,11]. Although systemic corticosteroids remain the cornerstone of therapy, recent reviews highlight the role of additional immunosuppressive or biologic agents in selected patients with multisystem disease [15]. Conventional immunomodulatory agents, including azathioprine and methotrexate, have been used as steroid-sparing therapies with variable success [11]. Notably, several case reports have documented favorable responses to TNF-α inhibitors, such as infliximab and adalimumab, with concurrent improvement in both cartilaginous inflammation and intestinal disease activity [4,5,14].

Collectively, the accumulating evidence supporting the efficacy of TNF-α inhibitors suggests that biologic therapy targeting shared inflammatory pathways may be particularly beneficial in patients with multisystem involvement or refractory disease. However, treatment decisions should be individualized, considering disease severity, organ involvement, and the potential risks associated with long-term immunosuppression.

From a clinical perspective, this case highlights several critical considerations. RP can rarely coexist with CD, and this association may be underrecognized due to the heterogeneous and episodic nature of RP. In patients with RP who develop new gastrointestinal symptoms or inflammatory arthritis—particularly during glucocorticoid tapering—clinicians should maintain a high index of suspicion for concomitant IBD rather than attributing symptoms solely to medication-related adverse effects. Early recognition of coexisting autoimmune conditions is crucial to avoid diagnostic delay and enable timely initiation of appropriate immunomodulatory therapy, thereby potentially improving clinical outcomes. Increased awareness of this rare coexistence may facilitate more comprehensive evaluation and optimized management strategies in patients presenting with multisystem inflammatory disease.

## Data Availability

The original contributions presented in this study are included in the article. Further inquiries can be directed to the corresponding author.
